# Prevalence and spatial distribution of Trematode cercariae in Vector Snails within different Agro-Ecological Zones in Western Kenya, 2016

**DOI:** 10.11604/pamj.2019.32.142.14418

**Published:** 2019-03-26

**Authors:** Maurice Omondi Owiny, Mark Odhiambo Obonyo, Peter Maina Gatongi, Eric Maurice Fèvre

**Affiliations:** 1 Kenya Field Epidemiology and Laboratory Training Programme, Ministry of Health, Kenyatta National Hospital Grounds, Nairobi, Kenya; 2 Moi University, School of Public Health, Eldoret, Kenya; 3 Institute of Infection and Global Health, University of Liverpool, Leahurst Campus, United Kingdom; 4 International Livestock Research Institute, Nairobi, Kenya

**Keywords:** Snails, cercariae, fasciola, schistosoma, infection, habitat

## Abstract

**Introduction:**

Freshwater vector snails' distribution, infection with cercariae, preferred habitat and possible trematodiases transmission foci is not well known in Western Kenya. We sought to determine the distribution and prevalence of infection of snails per agro-ecological zone and environmental factors in vector snail habitats.

**Methods:**

We conducted a cross-sectional survey from March, 2016 - May, 2016, harvested and identified snails using shell morphology, determined their infection with trematode cercariae using microscopy, used descriptive statistics to estimate the prevalence of infection and relationship between snail abundance and environmental factors.

**Results:**

We sampled a total of 1,678 vector snails from 47 sampling sites of which 42% were Lymnaeid, 23% Biomphalaria, 10% Bulinus, 22% Oncomelaniae and 2% Melanoides. Lower Midland I Ago-Ecological Zones had 44% of the snails and streams from springs had 41% of the snails. Overall, 26.5% (445/1678) (95% CI: 24.4 - 28.6) of the snails shed cercariae. Cercariae were found in 11 (23%) of the sites and in all zones. *F. gigantica* cercariae were shed by *L. natalensis, B. pfeifferi, B. sudanica*. Lakeshore had both *F. gigantica* and S. mansoni cercariae shed by *B. sudanica*. About 72% (1,202/1,678) of snails were found in water with a pH 6.5 - 7.5. Grass habitat had 54% (912/1,678) of the snails.

**Conclusion:**

Lymnaeid snails were present in all the zones, while streams from springs and near neutral habitats had most of the snails. Infection with trematode cercariae was noted in all the zones. Trematodiases control should be focused on all zones especially in freshwater streams and lakeshores.

## Introduction

Freshwater vector snails of medical and veterinary importance act as obligate intermediate hosts for parasitic trematodes [1, 2]. Vector snails play a vital role in the transmission, epidemiology and control of trematode infections, such as fascioliasis, a zoonotic food-borne trematode infection caused by parasitic trematodes, *Fasciola hepatica* and *Fasciola gigantica* [3] and Schistosomiasis that is caused by *Schistosoma mansoni* and Schistosoma haematobium [4-6]. Both Fascioliasis and Schistosomiasis are considered as neglected tropical diseases by the World Health Organization (WHO) since they affect some of the poorest of the poor populations in the world [7]. The chronic nature of trematode infections in the human and animal hosts makes it difficult to determine when and where transmission actually occurs [1, 3, 8, 9]. Investigation of freshwater vector snails provides vital information on active transmission foci for trematode infections [9, 10]. In Kenya, trematode infections are prevalent mostly in domestic livestock where they cause economic losses especially when the infected liver prevents trade and exportation of products of animal origin [11]. In Western Kenya it was estimated that total economic losses due to liver cirrhosis for a 10-year period was US$ 0.8 million [11, 12]. There is little information on trematode infections and the distribution of the snail vectors in Western Kenya and other parts of Africa too [3]. Busia County in Western Kenya has the conducive habitat for proliferation of vector snails and the trematode parasites that use them as intermediate hosts [13, 14], however, effective surveillance systems are lacking in veterinary and medical disease surveillance system in the region [15]. This study aimed at estimating prevalence of infection by identifying species of snails infected with trematode cercaraie in different Agro-Ecological Zones (AEZs) in Busia Kenya and to identify the environmental and physico-chemical factors that promote the presence of these snails in their habitats. By determining the distribution of the vector snails, our study findings could be used to identify possible disease transmission zones or foci for the trematode parasites. The knowledge on geographic distribution of the intermediate host snails could help improve efficiency of allocation of the available limited resources in control interventions to specific transmission foci.

## Methods

**Study design:** We conducted a cross-sectional study between March and May 2016 to investigate snails of veterinary and medical importance.

**Figure 1:**
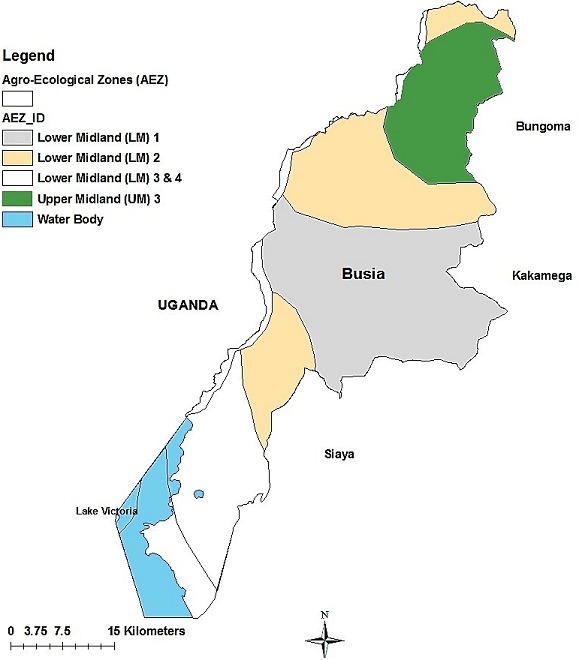
Study area showing the Agro-Ecological Zones (AEZs) in Busia, Kenya, 2016

**Study site:** The study was conducted in Busia County, in Western part of Kenya. Busia is close to Lake Victoria, has tropical humid climate, receives high rainfall throughout the year and has several rivers used for irrigation making it predominantly agricultural. Annual temperatures range from 17 to 30°C with mean annual temperatures ranging between 24°C and 26°C. Annual average precipitation is between 900mm and 1500mm distributed throughout two main rainy seasons with long rains between March and June and short rains between September and December. The area has four agro-ecological zones namely, lower mid-land (LM) made of LM 1, LM 2, LM 3 and 4 and upper midland (UM 3) [16-18]. The wetter LM 1 lies in the middle in Busia and Nambale Sub Counties while the LM 3 and 4 are found in the south-west in Budalangi and Samia Sub Counties. To the North-East lies Teso North Sub County in UM 3 (Figure 1).

### Operational definitions

**Agro-ecological zone:** Type of land and its suitability for different crops and combines data on soil, topography and climate.

**Lower midland zone:** Altitude range of 800 - 1500 meters, warm temperature with mean annual range of 21°C - 24°C, mean minimum temperature of about 14°C and suitable for growth of cotton, maize and rice.

**Upper midland zone:** Area with altitude ranging from 1300 - 1900 meters, mean annual temperature range of 18°C - 21°C, mean minimum temperature range of 11°C - 14°C and suitable for growth of Arabica coffee, maize.

**Spring/stream:** A stream originating from a freshwater spring.

**Lakeshore/swamp:** A swamp next to a freshwater lake.

**Dam/stream:** A stream originating from a dam.

**Borehole/stream:** A stream originating from a borehole.

**Pool/stream:** A stream originating from a pool of water.

**River/swamp:** A swamp next to a river.

**Swamp/stream:** A stream originating from a swamp.

**Well/stream:** A stream originating from a well.

**Sample size determination and assumptions:** We used Cochran formula (1977) to calculate a minimum sample size of 384 snails for each of the four agro-ecological zones giving a total of 1,536 snails for the whole study area with assumption of 50% prevalence of snail infection with cercariae (no available study in Kenya to reference), precision of 0.05 and Z-score of 1.96 for 95% confidence interval.

**Sampling technique and harvesting of snails:** Pre-study site visit was made to identify possible habitats and concentration of the snail vectors in each sampling site and AEZ. Sites to be sampled within the identified AEZs were selected on the basis of preliminary field observations on water contact points where people consistently went to collect water, wash clothes, bathe, swim, play, wash cars or water their livestock. Purposive selection of sampling sites ensured regional representation. We then randomly selected water bodies per AEZ depending on snail concentration in each site. The identified sites were mapped using Global Positioning System (GPS) and the geo-coordinates recorded appropriately. The snails were harvested from sites using dip scoop in areas with large water bodies or hand collection in areas with shallow water bodies. Sampling was extended to pastures near the water bodies. Sampling time was fixed at 15 minutes per location and performed between 8.00am and 11.00am. The presence and number of each snail species was recorded.

**Snail identification:** Snails from each site were appropriately labelled and transported in separate perforated plastic containers to the International Livestock Research Institute (ILRI) laboratory in Busia where they were processed. Species of snails were identified based on shell morphology by use of identification key developed by Mandahl-Barth for the identification of East and Central African snails of medical and veterinary importance [19].

**Identification of cercariae:** The snails were placed, proportionately based on species and sampling site, in groups of not more than 20 snails in flat-bottomed transparent vials containing water drawn from the ponds where snails were harvested. They were exposed to indirect sunlight for a maximum duration of four hours for cercariae shedding. The vials in which cercariae were not shed on the first exposure were re-exposed on the subsequent days until the fifth day. From the water in which snails were placed, 200 μl (0.2ml) of cercariae suspension was drawn. The suspension was mixed gently to allow for even distribution of cercariae before withdrawing aliquots of 5-10 of cercariae suspension from each vial. The aliquots were then placed in a petri dish and a sufficient amount of tap water added to cover the bottom of the petri dish. The cercariae in the petri dish were then killed by adding 1-2 drops of iodine solution and then let to stand for about one minute to allow the stained cercariae to settle to the bottom of the dish. By use of stereomicroscope at 8-40 times magnification, identification of cercariae was done by use of gross morphological characteristics, number and position of body suckers, shape and relative dimensions of the cercariae tail and the presence or absence of various specialized surface structures like stylet. The number and type of identified vector snails and trematode cercariae were recorded and grouped in appropriate tables.

**Determination of ecological characteristics:** At each sampling site, pH of water was measured by use of a multi-parameter water quality meter and temperature of water measured using industrial alcohol thermometer. The ambient temperature and type of vegetation cover were then recorded in a standard data abstraction tool.

**Statistical methods:** We calculated descriptive analysis to determine proportions of snails and prevalence of infection in different sites and agro-ecological zones using Epi Info™ 7.1.4.0 (CDC, Atlanta, GA, USA) and Microsoft Excel^®^ (Microsoft, Seattle, WA, USA). Mapping of the sampling sites was done using Arc GIS^®^ (ESRI, New York, Redlands, CA, USA) software. We calculated correlation coefficient to determine the relationship between snail abundance and the environmental and physico-chemical characteristics of the habitats.

## Results

**Figure 2:**
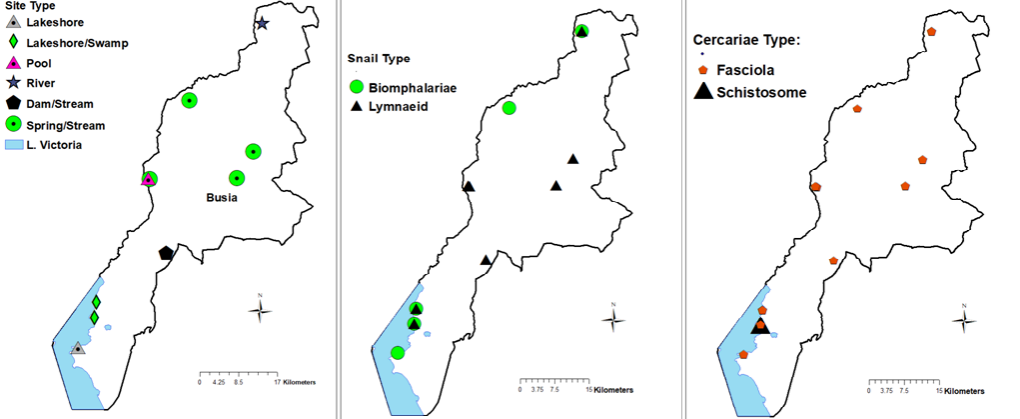
(A) sampling sites; (B) infected vector snails; (C) trematode cercariae in sampling sites, Busia, Kenya, 2016

**Figure 3:**
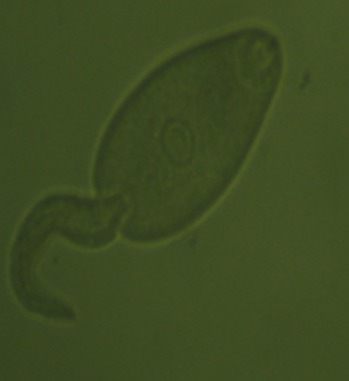
Fasciola cercaria shed by *L. natalensis*, Busia, Kenya, 2016

**Figure 4:**
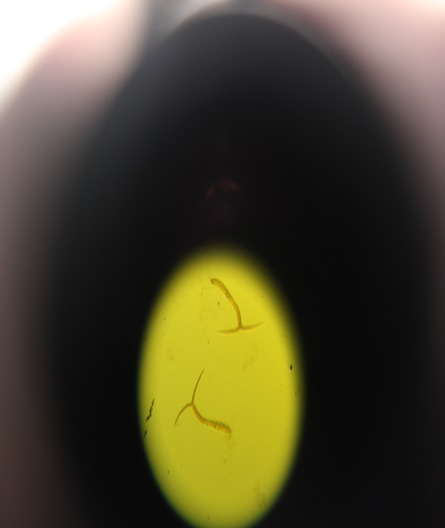
Schistosome cercaria shed by *B. pfeifferi*, Busia, Kenya, 2016

**Figure 5:**
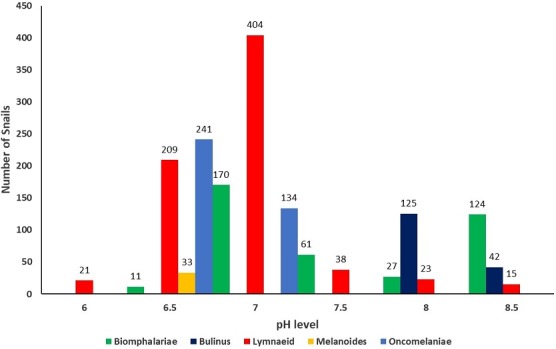
Distribution of vector snails by pH ranges, Busia, Kenya, 2016

A total of 1,678 vector snails of medical and veterinary importance and 559 prosobranch snails of little public health significance were collected from 47 sites in the study area. The sampling sites were distributed in the different AEZs with 18 (38%) in LM1, 16 (34%) in LM2, 7 (15%) in LM 3 and 4 and 6 (13%) in UM3. The major vector snail types included; Lymnaeid 710 (42%), Biomphalariae 393 (24%), Bulinus 167 (10%), Oncomelaniae 375 (22%) and Melanoides 33 (2%). Out of the lymnaeid type, *L. natalensis* accounted for 98.6% (700/710). Of the vector snails harvested 735 (44%) came from LM1 Zone and 319 (43.4%) of them belonging to the lymnaeid type (Table 1). Site types had varying snail numbers with streams originating from freshwater springs having the majority, 686 (41%) of the snails. Snails from 23.4% (11/47) (95% CI: 16.7 - 28.6) of the sites were infected with trematode cercariae. Infection was noted in all the Agro-Ecological Zones with LM 3 and 4 having majority of infected sites. The sites with infection were the lakeshore, swamps along the lakeshore, fresh water pools, fresh water river in the UM3, steams originating from dams and streams originating from water springs (Figure 2 A). Infected lymnaeid snails were found in all the AEZs while the majority of biomphalariae snails were found along the lakeshore (Figure 2 B). Fasciola cercariae were excreted by vector snails in all the AEZs with the schistosome cercariae being excreted by vector snails along the lakeshore (Figure 2 C). Overall, 26.5% (445/1678) (95% CI: 24.4 - 28.6) of the snails shed cercariae. *F. gigantica*cercariae (Figure 3) were shed by *L. natalensis*, *B. pfeifferi* and *B. sudanica*. In one site, a swamp near the shores of Lake Victoria, both *F. gigantica* and *S. mansoni* cercariae (Figure 4) were shed by *B. sudanica* vector snails harvested from the site. A majority of the vector snails (71.6%) were found at water pH between 6.5 and 7.5 while 10.8% of the vector snails were harvested from water with pH of 8.5 - 9.0. Only 22 (1.3%) of the snails were found in water with pH of 6 - 6.5 (Figure 5). The correlation coefficient between snail population and water pH was 0.22. Water habitat with grass harboured 912 (54.4%) of the snails while habitat with reeds harboured 196 (11.7%) of the snails.

**Table 1:** Vector snails genera harvested from different Agro-Ecological Zones, Busia County, Kenya, 2016 (N=1678)

AEZ	Lymnaeid (%)	Biomphalariae (%)	Bulinus (%)	Oncomelaniae (%)	Melanoides (%)	Total(% of N)
LM 1	319(45)	146(37)	0 (0)	270(72)	0 (0)	735(44)
LM 2	282(40)	105(27)	125(75)	44(12)	0 (0)	556(33)
LM 3,4	96(14)	131(33)	42(25)	0 (0)	33(100)	302(18)
UM 3	13(2)	11(3)	0 (0)	61(16)	0 (0)	85(5)

Key: AEZ: Agro-Ecological Zone, LM 1: Lower Midland I, LM 2: Lower Midland 2, LM 3 & 4: Lower Midland 3 and 4, UM III: Upper Midland 3

## Discussion

Food-borne trematodiases are infections with negative socio-economic and health impact on life of humans and animals. It is therefore important to identify sites and trematode species that exist in areas where the diseases are endemic. The chronic nature of the disease makes it difficult to detect early in both humans and animals, as a result, mapping of possible transmission foci for the diseases is key in putting preventive and control measures. The transmission foci are not well known in the western part of Kenya where the disease is common in livestock. Human infection with Fasciolosis and other trematodiases can occur in areas endemic with the vector snails and where the rural farming communities consistently use the same water source as their animals or ingest raw water-based vegetation that grows in contaminated areas as is common in western Kenya. We identified the possible transmission foci for Fasciolosis that could help in having targeted control and surveillance of trematode infections in human and animals. This study described the distribution of vector snails and the infective stages of the trematode cercariae. We demonstrated the existence of various species of vector snails of medical and veterinary importance in all agro-ecological zones of Busia County, with streams originating from springs and swamps near lakeshores being the preferred sites for most of the vector snails. Lymnaeid and Biomphalariae snail types shed trematode cercariae of the genera Fasciola and Schistosoma indicating the existence of infective stages of trematode in the study area. The findings of this study elucidated the presence of infected snails in fresh water springs where humans would not suspect to be polluted and as a result go about their day to day activities of herding and use of spring water for domestic use. Use of raw water as is common in the rural areas of Kenya pose great risk of infection with water borne diseases. Most herders were watering their animals in potentially contaminated spring and swamp water thereby exposing the animals and humans to infective stages of the trematodes. Humans were getting into contaminated water without any protective attire. The chronic nature of the trematode infection and the fact that trematode infections have similar clinical manifestations to other infectious diseases like malaria in human beings and Anaplasmosis in livestock, cases of misdiagnosis may ensue leading to wrong medication thereby exacerbating the disease. From the findings of this study, it would be wise to include trematode infections as differential diagnoses that should be ruled out in endemic areas.

Lymnaeid snails were widely distributed in all the agro-ecological zones and were the majority in LM 1 and LM 2. The other snail types, Biomphalariae, Bulinus, Oncomelaniae and Melanoides were only present in some of the zones. A majority of Biomphalariae were found in swamps near the lakeshore. This was in agreement with a study done by Opisa and others who found that *B. sudanica* were mostly found along the shores of lake Victoria and few in the inland sites [14]. Almost all lymnaeid snails were found in streams that originated from springs located away from the lakeshore. This was in line with similar findings in a study done in Venezuela where *L. truncatula* was found to prefer higher altitudes [20]. Howell and others also found a similar distribution in which populations of *L. natalensis* were high at lower altitude but decreased towards higher altitudes while *L. truncatula* was found mostly at higher altitude [15]. However, the findings in our study were in contrast to those from a study conducted on host snails in land and lake habitats of western Kenya that found ponds to be preferred by host snails for schistosomiasis [13]. In this study, Biomphalariae and Lymnaeid species were found to be infected with trematode cercariae. The *B. sudanica* species found in the swamp near the lakeshore were infected with both Fasciola gigantica and *Schistosoma mansoni* pointing to a co-existence of Schistosoma and Fasciola infection in the site. A study done in Egypt found that more than one trematode species could exist in one snail species that was more prevalent in that area, though the presence of one would lead to decrease in the others especially where there was competition [21]. *B. pfeifferi* species in LM 1 and UM 3 were infected with *Fasciola gigantica* meaning that *F. gigantica* could be found both at low altitude and at high altitude. In addition, *F. gigantica* cercariae were shed by *L. natalensis* snails in LM 1, LM 2 and UM 3. This was comparable to findings in a study conducted in Tanzania in 2015 in which the authors noted that *F. gigantica* was present at both high and low altitude [22]. The presence of *F. gigantica* in almost all the agro-ecological zones in Busia means that the humans and animals living in the area can contract Fasciolosis from the natural water sources used for domestic purposes in the area. The vector snails were found to prefer water habitats with grass and almost neutral pH range. Infected snails were largely found in habitats with near-neutral water pH and in areas where there was significant human and animal activities. Humans and animals tend to prefer water with near neutral pH and if such type of water is consumed raw may lead to trematode infection. A study conducted in Nigeria found that snails were absent in habitats with high alkalinity [23], a result which is similar to our findings where the presence of snails was recorded at low alkalinity and not at high alkinity. However, Salawu *et al* and Dida *et al* found no positive correlation between populations of *L. natalensis* and pH [24, 25].

Vegetation cover influences the abundance of freshwater snails since they use it for cover, attachment, egg laying and food as was reported in a study on land and lake habitats where plants like hippo grass and hyacinth were preferred by Biomphalariae species [24]. In this study, grass was identified as preferred vegetation for almost all vector snails with all *B. sudanica* being harvested from grass in swamps. Humans and animals using contaminated water and vegetation near streams originating from springs and swamps next to lakeshores could contract Trematodiases in endemic areas especially whenever they get in infected water with open wounds and without protective attire. The use of purposive selection of sampling sites may have led to areas with infective stages of trematodes being left out in this study thus having a possibility of not giving actual prevalence of vectors snails and trematode infection. We harvested snails towards the end of dry season in March, 2016 and at the beginning of wet season of April, 2016. Snail populations vary with weather since the number increases during wet weather and decreases during dry season. This study results may have been unduly influenced by the weather at the time of snail harvesting resulting in high figures in areas that were sampled during wet weather. Inaccessibility to some of the areas meant that such sites were left out during the sampling period and this may have led to uneven distribution of sites in some AEZs. Despite the limitations, this study indicated that the distribution of freshwater vector snails was varied across the AEZ's with all the zones having varied numbers of snails. The streams originating from springs seemed to provide a conducive habitat for the snails due to fresh water from the springs. Presence of vector snails in all of the zones pointed to presence of possible transmission foci for Fasciolosis and other foodborne trematodiases. People and animals using water and pasture from these sites in Busia could contract these parasitic infections. A serological survey should be carried out in the study area to ascertain the existence of trematode infections in animals and human. More studies are needed on molecular characterization of the vector snails and the trematode cercariae in the study area and to explore the possibility of co-infection in humans and animals in areas where more than one type cercariae were shed. Study to identify environment-friendly ways of controlling the population of snail in the study area should be encouraged as a way of prevention of trematode infections. In addition, public health education and awareness should be initiated in the study area to sensitize the local community on the dangers of using raw water from the springs and swamps and the need to use protective equipment like gum boots in potentially contaminated water bodies.

## Conclusion

Community members and their animals may use water that is inhabited by infected snails thus pointing to possibility of infection from the trematode cercariae that may be shed by the vector snails in water destined for domestic use. We therefore recommend that, control of trematode parasitic infections should be targeted at all the AEZ's with emphasis placed on the areas that border the lake and those with streams flowing from freshwater springs. Communities using water bodies in Busia should be educated and made aware of potential risk to them and their livestock of contracting trematode infections. People who frequent and get in contact with water from streams and swamps should be advised to use protective gear like gum boots and gloves especially for those with open wounds and skin lesions. Water from potentially infected sources should be treated before domestic use. Community members should avoid consuming raw vegetation and washing of fruits or vegetables using water from areas with high possibility of infection. Use of environment-friendly molluscicides to control vector snails, water treatment chemicals to treat water for domestic use and regular deworming of human beings and livestock should be encouraged.

### What is known about this topic

- Fresh water snails are obligate vectors for completion of life cycle of trematodes which pose public health problems in some of the poorest communities;
- Food borne trematodiases, which are neglected tropical diseases, affect both humans and animals.

### What this study adds

- Infective stages of trematodes are prevalent in fresh water originating from springs in Busia, Kenya;
- Vector snails are found in all the agro-ecological zones in Busia, Kenya with lakeshore and fresh water springs having the majority of the snails;
- More than one species of trematode cercariae could be shed by one vector snail leading to possible comorbidity.

## Competing interests

The authors declare no competing interests.
